# A Microbiome-Derived Peptide Induces Apoptosis of Cells from Different Tissues

**DOI:** 10.3390/cells10112885

**Published:** 2021-10-26

**Authors:** Haruko Saiki, Yuko Okano, Taro Yasuma, Masaaki Toda, Atsuro Takeshita, Ahmed M. Abdel-Hamid, Valeria Fridman D’Alessandro, Tatsuki Tsuruga, Corina N. D’Alessandro-Gabazza, Kan Katayama, Masahiko Sugimoto, Hajime Fujimoto, Keiichi Yamanaka, Tetsu Kobayashi, Isaac Cann, Esteban C. Gabazza

**Affiliations:** 1 Department of Pulmonary and Critical Care Medicine, Faculty and Graduate School of Medicine, Mie University, Tsu 514-8507, Mie, Japan; harusakusa@yahoo.co.jp (H.S.); y.o.spriggan@gmail.com (T.T.); genfujimoto1974@yahoo.co.jp (H.F.); kobayashitetsu@hotmail.com (T.K.); 2 Department of Immunology, Faculty and Graduate School of Medicine, Mie University, Tsu 514-8507, Mie, Japan; yu.higako@gmail.com (Y.O.); t-yasuma0630@clin.medic.mie-u.ac.jp (T.Y.); t-masa@doc.medic.mie-u.ac.jp (M.T.); johnpaul0114@yahoo.co.jp (A.T.); immunol@doc.medic.mie-u.ac.jp (V.F.D.); dalessac@clin.medic.mie-u.ac.jp (C.N.D.-G.); 3 Department of Diabetes, Endocrinology and Metabolism, Faculty and Graduate School of Medicine, Mie University, Tsu 514-8507, Mie, Japan; 4 Department of Botany and Microbiology, Faculty of Science, Minia University, El-Minia 61519, Egypt; ahetta@illinois.edu; 5 Carl R. Woese Institute for Genomic Biology (Microbiome Metabolic Engineering), University of Illinois at Urbana–Champaign, Urbana, IL 61801-3633, USA; icann@illinois.edu; 6 Department of Cardiology and Nephrology, Faculty and Graduate School of Medicine, Mie University, Tsu 514-8507, Mie, Japan; katayamk@clin.medic.mie-u.ac.jp; 7 Department of Ophthalmology, Faculty and Graduate School of Medicine, Mie University, Tsu 514-8507, Mie, Japan; sugmochi@clin.medic.mie-u.ac.jp; 8 Department of Dermatology, Faculty and Graduate School of Medicine, Mie University, Tsu 514-8507, Mie, Japan; yamake@clin.medic.mie-u.ac.jp; 9 Departments of Animal Science, and Microbiology, The University of Illinois at Urbana-Champaign, Urbana, IL 61801-3633, USA

**Keywords:** corisin, apoptosis, organ fibrosis, parenchymal cells, different tissue

## Abstract

Apoptosis is a programmed cell death involved in embryogenesis and tissue homeostasis under physiological conditions. However, abnormalities in the process of apoptosis are implicated in the pathogenesis of various diseases. The human microbiota may release products that induce apoptosis of host cells. We recently identified a novel microbiome-derived peptide called corisin that worsens lung fibrosis by inducing apoptosis of lung epithelial cells. We hypothesized that corisin and a corisin-like peptide might also induce apoptosis of cells from different tissues. We cultured podocytes, renal tubular epithelial cells, keratinocytes, retinal and intestinal cells treated with corisin and evaluated apoptosis by flow cytometry and Western blotting. Although at different grades, flow cytometry analysis and Western blotting showed that corisin and a corisin-like peptide induced apoptosis of podocytes, keratinocytes, tubular epithelial cells, retinal, and intestinal cells. In addition, we found that corisin synergistically enhances the proapoptotic activity of transforming growth factor-β1 on podocytes. In conclusion, these results suggest that corisin and corisin-like peptides may play a role in the pathogenesis of disease in different organs by promoting apoptosis of parenchymal cells.

## 1. Introduction

Apoptosis is a programmed cell death with active participation in embryogenic development and maintenance of tissue homeostasis under physiological conditions [1]. It may be extrinsic or intrinsic, depending on the activation pathway. Extrinsic apoptosis is triggered by extracellular stress signals detected by transmembrane death receptors [1]. Intrinsic apoptosis is initiated by the mitochondrion, activated by the withdrawal of growth factors, hypoxia, oxidative stress, and finely regulated by the B-cell lymphoma 2 (Bcl-2) family of proteins [2,3]. Abnormalities in the process of apoptosis are implicated in the mechanism of several diseases. Defective apoptosis occurs in malignant tumors [4], autoimmune disorders [5], and infection by intracellular pathogens [6], whereas excessive apoptosis of organ parenchymal cells contributes to the pathogenesis of neurodegenerative diseases (e.g., Alzheimer’s disease [7], amyotrophic lateral sclerosis [8], Parkinson’s disease [9]), organ fibrosis (chronic kidney disease [10], liver cirrhosis [3], idiopathic pulmonary fibrosis [11], diabetic retinopathy [12], atopic dermatitis [13]), or vascular diseases (ischemic heart disease [14], cerebral ischemia [15], atherosclerosis [16]). The causative factors of apoptosis dysregulation are unclear. However, recent studies implicated the host microbiome in pathologic apoptosis [17,18,19]. 

The microbiome represents the complete genetic material harbored by microorganisms or microbiota that inhabit niches (e.g., skin, gut, lungs, urinary tract, eyes) of the human body [1]. The physiological function of the human microbiota is essential for nutrient degradation, biosynthesis of vitamins and amino acids, priming, and development of the immune system [1]. However, dysbiosis of the microbiome has been linked to disease development [20]. The mechanisms are not entirely clear; however, apoptosis of host cells has been reported to play a role. For example, oral dysbiosis is associated with enhanced apoptosis and disease progression in patients with chronic periodontitis and increased circulating levels of trimethylamine-n-oxide, a gut microbiota-dependent metabolite, promotes apoptosis of vascular smooth muscle cells and atherosclerosis in patients with chronic kidney disease [17,18,19]. In addition, we recently identified a peptide called corisin, released by *Staphylococcus nepalensis* strain CNDG from a fibrotic lung tissue, and this peptide induces apoptosis of alveolar epithelial cells and acute exacerbation of pulmonary fibrosis [21]. A significantly high level of the proapoptotic peptide was also detected in patients with idiopathic pulmonary fibrosis with acute exacerbation compared to patients without acute exacerbation and healthy controls, suggesting the pathogenic implication of corisin in the human lung fibrotic disease [21]. The evolutionary evaluation showed that the corisin sequence is highly conserved in the *Staphylococcus* *genus*, highlighting its functional significance [21]. 

In the present study, we hypothesized that corisin also induces apoptosis of cells from different tissues. To test our hypothesis, we cultured cell lines from different tissues with corisin and also a corisin-like peptide from *S. haemolyticus* and assessed the capacity to induce apoptosis in each cell line. Here, we report our findings that show that the corisin and corisin-like peptides trigger apoptosis in cell lines derived from multiple human tissues.

## 2. Materials and Methods

### 2.1. Reagents

The clear cell renal carcinoma (Caki-2) cell line derived from the epithelium of the proximal tubules, the in vitro spontaneously transformed aneuploidy immortal keratinocyte (HaCaT) cell line from adult human skin, the spontaneously arising retinal pigment epithelial (ARPE-19) cell line, the colon carcinoma (Caco-2) cell line and the normal human small intestinal epithelial cell-6 (HIEC-6) were obtained from the American Type Culture Collection (Manassas, VA, USA. RPMI 1640 medium was purchased from Sigma-Aldrich (St. Louis, MO, USA) and fetal bovine serum (FBS) from Bio Whittaker (Walkersville, MD, USA). L-glutamine, penicillin, and streptomycin were from Invitrogen (Carlsbad, CA, USA). The scrambled peptide (NRVYNGPAASPVSEQMPIN), the corisin (IVMPESSGNPNAVNPAGYR), and the corisin-like peptide (IVMPESGGNPNAVNPAGYR) were synthesized and provided by Peptide Institute Incorporation (Osaka, Japan). Transforming growth factor (TGF)β1 was purchased from the R&D System (Minneapolis, MN, USA).

### 2.2. Cell Culture

Caki-2 cells, Caco-2 cells, and immortalized human podocyte (ihPOD) were cultured in RPMI 1640 medium, HaCaT cells in high-glucose Dulbecco’s Modified Eagle Medium (DMEM), ARPE-19 cells in DMEM/F12 medium, HIEC-6 cells in DMEM medium. Each medium was supplemented with 10% fetal calf serum (FCS), 0.03% (w/v) L-glutamine, 100 IU/mL penicillin, and 100 μg/mL streptomycin. The cells were cultured in a humidified, 5% CO_2_ atmosphere at 37 °C. The experiments were undertaken 10 days after seeding, when cells became differentiated.

### 2.3. Evaluation of Cell Apoptosis

The cells were cultured up to subconfluency. The cells were then washed and cultured overnight in an FCS-free medium. The cells were stimulated with 10 µg/mL of scrambled peptide, corisin, or corisin-like peptide and cultured for 48 h before collecting the cells for apoptosis evaluation by flow cytometry. To evaluate cleavage of caspase-3 by Western blotting, the cells were cultured and stimulated similarly with each peptide for 24 h before cell harvesting. To evaluate the synergism of corisin with TGFβ1, podocytes were cultured in the presence of both TGFβ1 and corisin for 48 h before evaluating apoptosis. As a control, we performed the same experiment, but with corisin replaced with its scrambled peptide. 

### 2.4. Flow Cytometry Analysis

The cells were analyzed for apoptosis by flow cytometry (FACScan, BD Biosciences, Oxford, UK) after staining with fluorescein-labeled annexin V and propidium iodide (FITC Annexin V Apoptosis Detection Kit with PI, Biolegend, San Diego, CA, USA).

### 2.5. Western Blotting

The cells were washed with ice-cold phosphate-buffered saline and treated with radioimmunoprecipitation assay (RIPA) lysis buffer supplemented with protease/phosphatase inhibitors. After cell centrifugation, the protein concentration was determined using the Pierce BCA protein assay kit (Thermo Fisher Scientific Incorporation, Waltham, MA, USA). Cellular lysate protein was mixed with Laemmli sample buffer, applied to a sodium dodecyl sulfate-polyacrylamide gel, and electrophoresis was run. After electrophoretically transferring the proteins from the gel to nylon membranes, Western blotting was performed using anti-cleaved caspase-3 or anti-β-actin antibody (Cell Signaling, Danvers, MA, USA) as previously described [20]. The intensity of the bands was quantified by densitometry using the public domain NIH ImageJ program (wayne@codon.nih.gov; Wayne Rasband, NIH, Research Service Branch).

### 2.6. Evaluation of Reactive Oxygen Species during Corisin-Induced Apoptosis

Caco-2 cells were pre-incubated with or without N-acetyl-l-cysteine, a reactive oxygen species (ROS) inhibitor, for 1 h, treated with 10 μg/mL of corisin or scrambled peptide for 48 h then apoptosis was evaluated by flow cytometry. To detect cellular ROS, Caco-2 cells (2 × 10^5^ cells/well) cultured in a 12-well plate were treated with 5 μM corisin or scrambled peptide for 48 hrs. Cells were washed with phosphate-buffered saline (PBS) and incubated with RPMI-1640 medium containing 20 μM 2′,7′-dichlorofluorescin diacetate (DCFDA) (R&D Systems, Minneapolis, MN, USA) for 20 min in the dark at 37 ºC. Fluorescence intensity of DCF was detected using a BD FACScan flow cytometer. Data were analyzed using the BD CellQuest software.

### 2.7. Mitochondrial Membrane Potential Assay

Loss of mitochondrial membrane integrity was measured using the fluorescent dye JC-1 (5,5′, 6,6′-tetrachloro-1,1′, 3,3′-tetraethyl tetrethyl benzimidalyl carbocyanine iodide) from Dojindo (Kumamoto, Japan). Changes in mitochondrial membrane potential were expressed as a ratio of red (585 nm) to green (530 nm) fluorescence. In the assay, Caco-2 cells were treated with 10 μg/mL of corisin or scrambled peptide for 48 h, and then cells were stained with 2 μM JC-1 for 20 min at 37 °C. JC-1 fluorescence was detected using BD FACScan flow cytometry, and data were analyzed using BD CellQuant software.

### 2.8. Statistical Analysis

Data were expressed as the mean ± standard deviation of the means (S.D.). The statistical difference between variables was calculated by analysis of variance (ANOVA) with Newman-Keuls’ test. Statistical analyses were performed using the GraphPad Prism version 7.0 (GraphPad Software, San Diego, CA, USA). A *p* < 0.05 was considered statistically significant.

## 3. Results

### 3.1. Differential Proapoptotic Activity of Corisin and Corisin-like Peptide on Cells from Different Tissues

**Figure 1:**
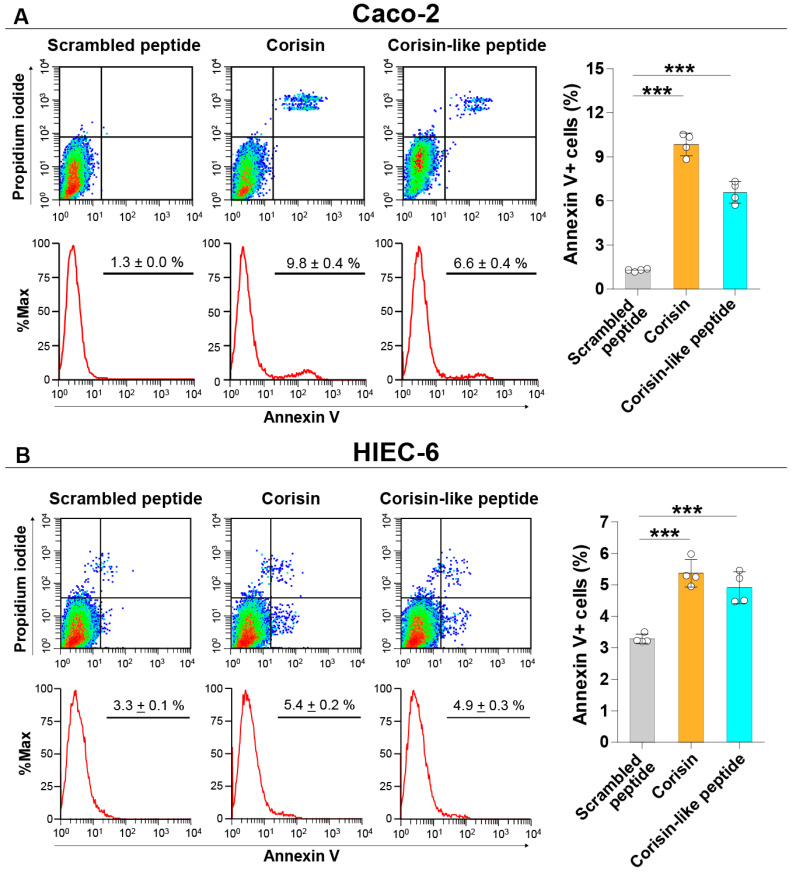
Corisin and corisin-like peptide induce apoptosis of intestinal cells. Caco-2 (**A**) and HIEC-6 (**B**) cells were cultured in the presence of corisin or corisin-like peptides for 48 h, and apoptosis was evaluated by flow cytometry. Data are expressed as the mean ± S.D. Statistical analysis by ANOVA with Newman-Keuls’ test. *** *p* < 0.001.

**Figure 2:**
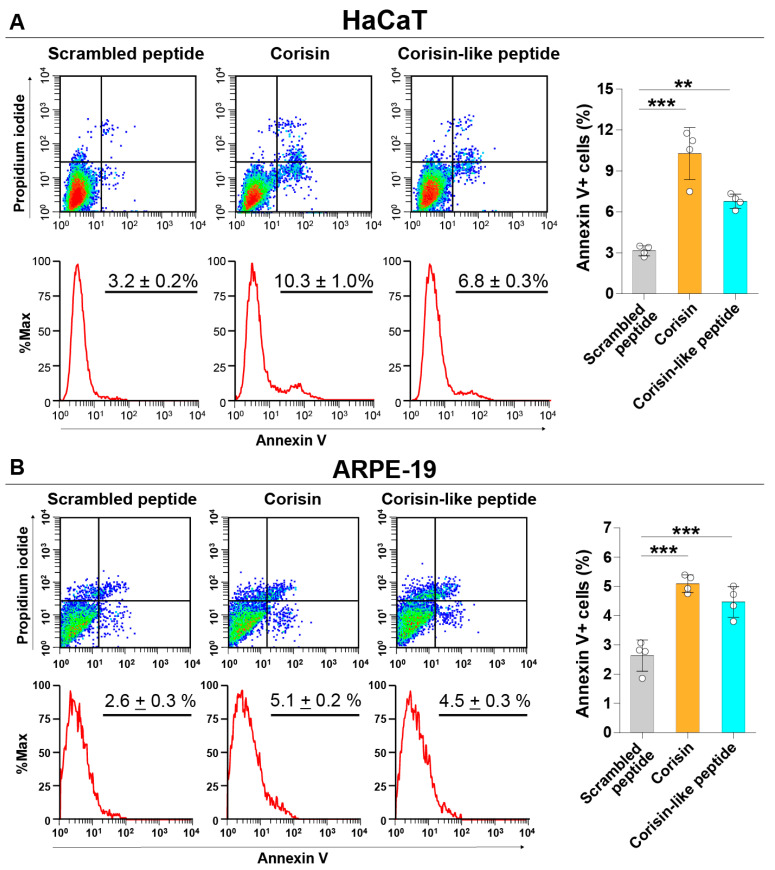
Corisin and corisin-like peptides induce apoptosis of keratinocytes and retinal cells. HaCaT (**A**) cells and ARPE-19 cells (**B**) were cultured in the presence of corisin or corisin-like peptide for 48 h, and apoptosis was evaluated by flow cytometry. Data are expressed as the mean ± S.D. Statistical analysis by ANOVA with Newman-Keuls’ test. ** *p* < 0.01; *** *p* < 0.001.

**Figure 3:**
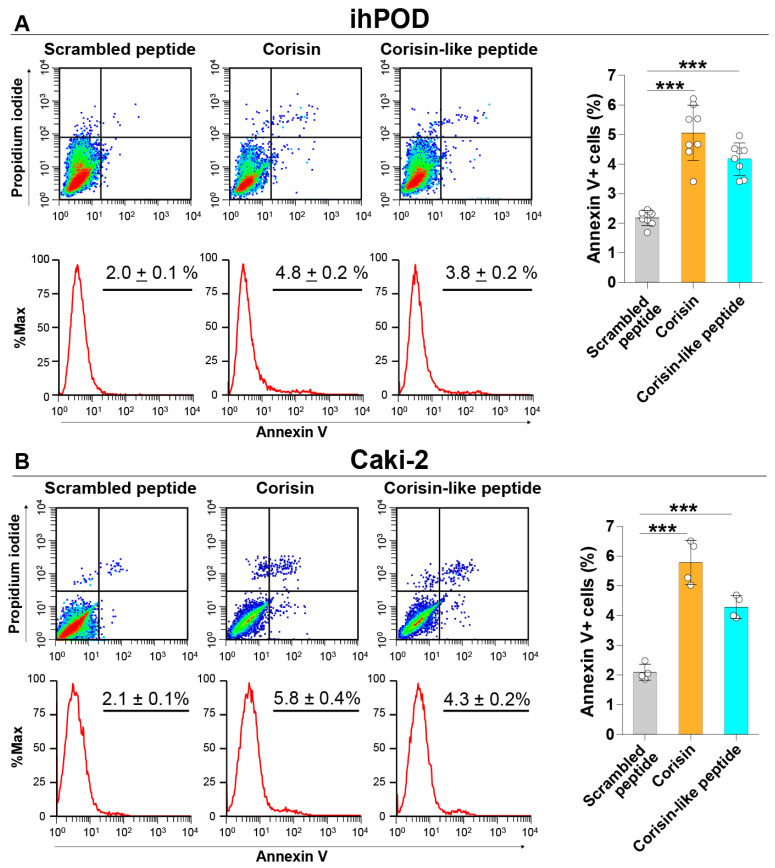
Corisin and corisin-like peptide induce apoptosis of podocytes and renal tubular epithelial cells. Podocytes (**A**) and Caki-2 cells (**B**) were cultured in the presence of corisin or corisin-like peptide for 48 h, and apoptosis was evaluated by flow cytometry. Data are expressed as the mean ± S.D. Statistical analysis by ANOVA with Newman-Keuls’ test. *** *p* < 0.001.

**Figure 4:**
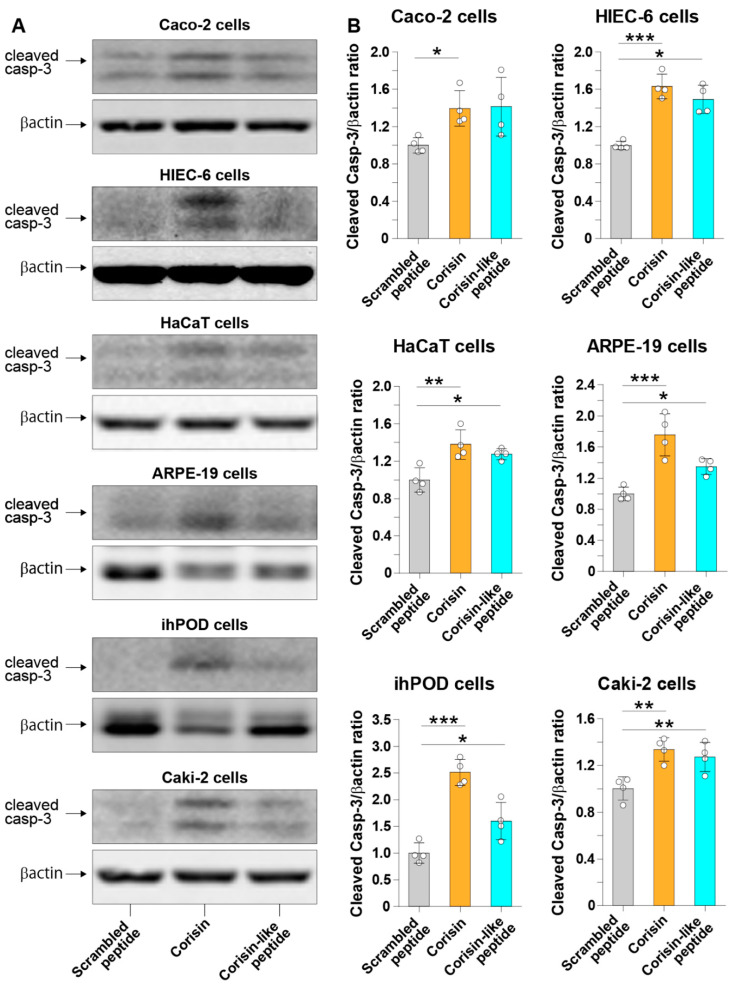
Cleavage of caspase-3 in cells treated with corisin or corisin-like peptide. Colon (Caco-2) epithelial cells, small intestinal (HIEC-6) cells, keratinocytes (HaCaT cells), retinal (ARPE-19) cells, podocytes (ihPOD), and renal tubular epithelial (Caki-2) cells were cultured in the presence of corisin or corisin-like peptide for 24 h, and apoptosis was evaluated by Western blotting (**A**). Densitometry analysis was performed using ImageJ (**B**). Data are expressed as the mean ± S.D. Statistical analysis by ANOVA with Newman-Keuls’ test. * *p* < 0.05, ** *p* < 0.01, *** *p* < 0.001.

Corisin and corisin-like peptides significantly induced apoptosis of cultured colon epithelial (Caco-2) cells and small intestinal (HIEC-6) epithelial cells compared to the scrambled peptide. The proapoptotic activity of the corisin-like peptide, which differs in one amino acid from corisin (7S→7G), is slightly weaker than corisin (Figure 1A,B). Corisin and corisin-like peptides also significantly induced apoptosis of keratinocytes (HaCaT) and retinal (ARPE-19) cells compared to the scrambled peptide (Figure 2A,B). Although at less degree than Caco-2 and HaCaT cells, podocytes (ihPOD) and renal tubular (Caki-2) cells also underwent apoptosis in the presence of corisin or corisin-like peptide (Figure 3A,B). Evaluation of caspase-3 activity showed a significantly increased cleavage of caspase-3 in all cells treated with corisin and corisin-like peptides compared to cells treated with scrambled peptides, indicating activation of the apoptotic pathway (Figure 4A,B).

### 3.2. Corisin Potentiates the Proapoptotic Activity of TGFβ1 on Podocytes

**Figure 5:**
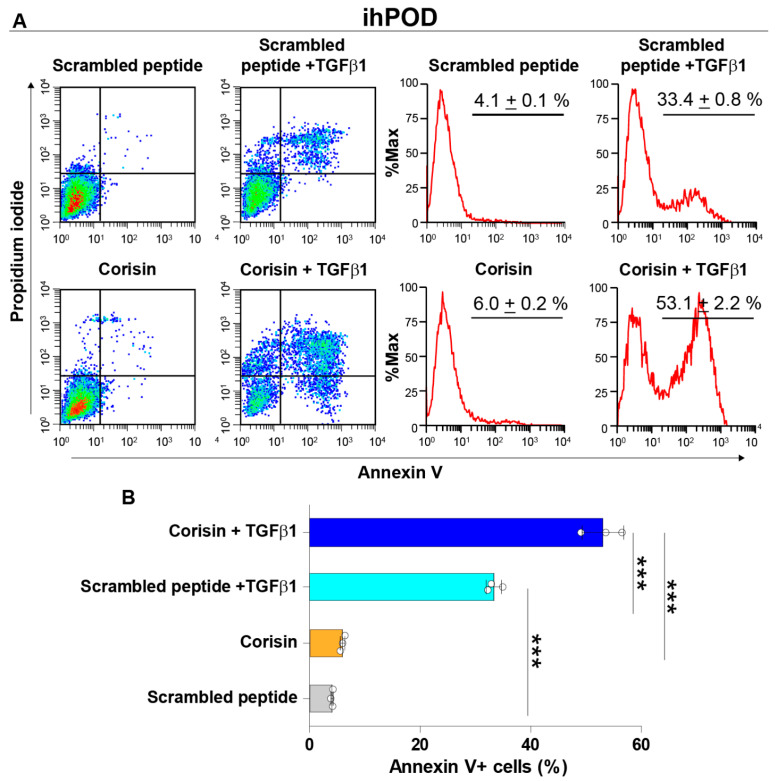
Corisin potentiates the proapoptotic activity of transforming growth factor-β1 synergistically. Podocytes were cultured in the presence of scrambled peptide alone, corisin alone, or TGFβ1 in combination with scrambled peptide or corisin for 48 h and the degree of apoptosis was evaluated by flow cytometry (**A**). The number of cells was expressed in percentage (**B**). Data are expressed as the mean ± S.D. Statistical analysis by ANOVA with Newman-Keuls’ test. *** *p* < 0.001.

The podocytes were cultured up to subconfluency, serum-starved, and then treated with a combination of TGFβ1 (5 ng/mL) and corisin (5 µg/mL) for 48 h before cell harvesting to assess apoptosis. Corisin alone induced weak apoptosis of podocytes, but it significantly potentiated the apoptotic activity of TGFβ1 on podocytes (Figure 5A,B).

### 3.3. Increased Generation of Reactive Oxygen Species and Loss of Mitochondrial Membrane Integrity during Apoptosis Induced by Corisin

**Figure 6:**
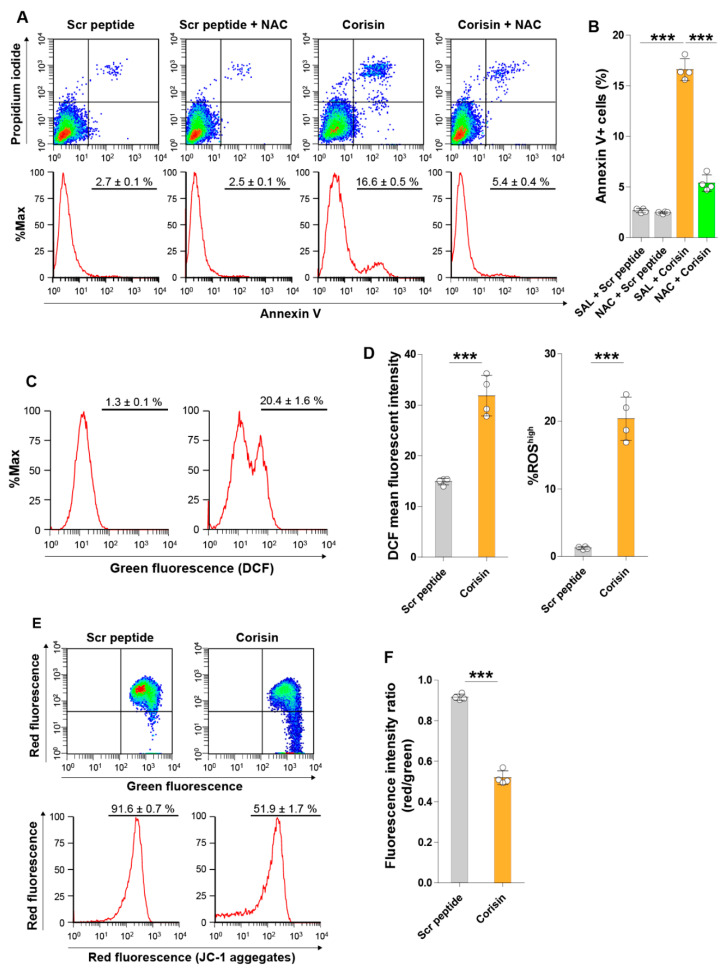
Treatment of intestinal cells is associated with increased generation of reactive oxygen species (ROS) and mitochondrial membrane depolarization. Caco-2 cells were pre-incubated with or without N-acetyl-l-cysteine and then treated with 10 μg/mL of corisin or scrambled peptide before evaluating apoptosis by flow cytometry (**A**,**B**). To detect cellular ROS, Caco-2 cells were treated with 5 μM corisin or scrambled peptide, washed with phosphate-buffered saline, incubated with medium containing 2′,7′-dichlorofluorescin diacetate (DCFDA) and then evaluated by flow cytometry (**C**,**D**). The JC-1 dye was used to evaluate mitochondrial membrane integrity. Caco-2 cells were treated with corisin or scrambled peptide, and then cells were stained with JC-1 before assessing by flow cytometry (**E**,**F**). Data are expressed as the mean ± S.D. Statistical analysis by ANOVA with Newman-Keuls’ test. *** *p* < 0.001.

N-Acetyl-l-cysteine, an inhibitor of reactive oxygen species, significantly suppressed apoptosis induced by corisin in Caco-2 cells compared to controls (Figure 6A,B). We also measured the generation of ROS using the ROS-sensitive DCFDA dye. There was a significantly increased ROS accumulation in cells treated with corisin compared to scrambled peptide-treated cells (Figure 6C,D). In addition, evaluation of the mitochondrial membrane potential using the JC-1 dye disclosed a significant reduction in the red to green fluorescence intensity ratio indicating mitochondrial membrane depolarization in Caco-2 cells treated with corisin compared to cells treated with scrambled peptide (Figure 6E,F). 

## 4. Discussion

This study shows that microbiome-derived peptides induce apoptosis of cells from different tissues and that corisin enhances the proapoptotic activity of TGFβ1 on podocytes synergistically. 

Corisin was originally identified in the culture supernatant of *S. nepalensis* isolated from the fibrotic lung tissue of transgenic mice with lung-specific overexpression of the human TGFβ1 gene [21]. Corisin is a fragment of bacterial transglycosylase, and its sequence is well conserved among microbes of the genus *Staphylococcus* [21]. Staphylococci are important members of the human microbiota [22]. Therefore, it is reasonable to speculate that in addition to the lungs, corisin production also occurs in other microbiota niches of the body and that it is involved in the pathological states of other organs. A mounting body of evidence showed that increased death of parenchymal cells plays a critical role in the pathogenesis of chronic kidney disease [10], chronic dermatitis [13], intestinal bowel disease [23], and diabetic retinopathy [12]. However, the causative factor of excessive cell death and whether the microbiome plays a role in the mechanism remain unclear. In the present study, we found that corisin induces apoptosis of keratinocytes, podocytes, renal tubular epithelial cells, intestinal and retinal cells, suggesting the potential pathogenic implication of corisin in the mechanism of cell death observed in intractable diseases. In line with this observation, the culture of intestinal cells in the presence of corisin was associated with increased ROS generation and loss of mitochondrial membrane integrity.

We previously reported that low levels of corisin can be detected by enzyme immunoassay in the bronchoalveolar lavage fluid of healthy subjects and the lung tissue by Western blotting, showing the existence of a baseline level of corisin released from the microbiome under physiological conditions [21]. Being a recently discovered proapoptotic factor, the physiological function of corisin or corisin-like peptides is currently unknown. However, we hypothesized that under pathological conditions in the presence of enhanced local levels of other proapoptotic factors such as TGFβ1 or tumor necrosis factor-α, even low concentrations of corisin may be sufficient to trigger cell death [24]. To test this hypothesis, we cultured podocytes and compared the degree of apoptosis induced in the presence of scrambled peptide, corisin alone, and in the presence of both TGFβ1 and corisin or scrambled peptide. The addition of corisin synergistically increased the degree of podocytes apoptosis induced by TGFβ1 alone plus scrambled peptide. Existing data indicate that TGFβ1 induces proliferation of fibroblasts, deposition of extracellular matrix, and podocytes apoptosis in chronic glomerulosclerosis. Therefore, the current study results suggest that corisin may further potentiate the detrimental effect of TGFβ1 in kidney fibrosis. 

In brief, this study shows that microbiome-derived peptides induce apoptosis of cells from different tissues and that corisin enhances the proapoptotic activity of TGFβ1 on podocytes synergistically. Our observation that corisin is stable in the body fluid further suggests that irrespective of where it is produced, (e.g., lung, intestinal, urinal tract) corisin has the potential to induce some form of systemic apoptosis, although in many tissues it may be low grade in nature.

## 5. Patents

C.N.D.-G., E.C.G. and I.C. have issued a patent on the apoptotic peptides described in this study and anticorisin monoclonal antibodies. 

## Data Availability

All data obtained during the current study are available from the corresponding author upon reasonable request.
